# Cultivating resilience and adaptability through hands-on One Health

**DOI:** 10.1080/16549716.2025.2478694

**Published:** 2025-03-19

**Authors:** Phaedra Henley, Anselme Shyaka

**Affiliations:** Center for One Health, University of Global Health Equity, Kigali, Rwanda

**Keywords:** Global health, education, experiential learning, interdisciplinary, Equity

## Abstract

The University of Global Health Equity‘s (UGHE) One Health Field School (OHFS) in Rwanda exemplifies an experiential approach to education that integrates human, animal, and environmental health. This field-based program engages students in real-world settings such as abattoirs, health centers, and mining sites to confront pressing public health issues, from zoonotic diseases and antimicrobial resistance to food safety and environmental health. Following Kolb's experiential learning model, OHFS equips graduate students to observe, analyze, and apply solutions to complex health challenges, fostering adaptability, resilience, and collaborative problem-solving skills. By bridging classroom theory with practical application, OHFS cultivates leadership and a holistic understanding of health, preparing graduates to navigate the interconnected global health landscape. Through this innovative approach, UGHE aims to train a generation of health professionals capable of addressing crises such as climate change, biodiversity loss, and emerging infectious diseases at the human–animal–environment interface.

## Paper Context

- **Main findings:** The University of Global Health Equity’s One Health Field School effectively integrates experiential learning with interdisciplinary collaboration to prepare students for complex global health challenges. By engaging students in real-world settings, the program fosters adaptability, resilience, and leadership in addressing interconnected health issues at the human-animal-environment interface.
- **Added knowledge:** The One Health Field School bridges the gap between theoretical knowledge and practical application, emphasizing interdisciplinary collaboration at the human–animal–environment interface. It builds capacity in future global health professionals to design and implement solutions for real-world health challenges, demonstrating the critical value of field-based, experiential learning in higher education.
- **Global health impact for policy and action:** The One Health Field School serves as a model for interdisciplinary education, promoting an integrated One Health approach. It informs global health policies by emphasizing the importance of equipping professionals with practical skills and holistic perspectives to address emerging health crises such as climate change, biodiversity loss, and food security.

## Background

As the world faces unprecedented challenges from climate change, biodiversity loss, and increasing health disparities, the need for an integrated approach to health has never been more critical. Emerging global health threats such as zoonotic diseases, which account for 60% of emerging infectious diseases [1] and cause 2.4 billion cases of infections and 2.2 million deaths annually [2], and foodborne diseases that have a global burden that rivals malaria, HIV/AIDS, and tuberculosis underscore the urgency of this need [3]. For example, the Marburg Virus outbreak in Rwanda, with confirmed 66 cases, including 15 deaths, comes as a reminder about the need to offer training programs that bring together multisectoral learners [4].

These programs should not only equip participants with holistic perspectives on the human–agriculture–wildlifeenvironment interface but also cultivate adaptability and resilience – qualities essential for navigating and addressing the complex challenges at the science–implementation–policy interface. Adaptability, the capacity to adjust to new conditions and challenges, enhances the ability to navigate uncertainty, while resilience, the ability to recover from adverse events and maintain functionality, ensures stability. Together, they form the foundation for addressing interconnected threats at the human–animal–environment interface, allowing learners and systems to absorb shocks, adapt to change, and sustain long-term health outcomes.

The University of Global Health Equity (UGHE) in Rwanda addresses these issues through its innovative One Health Field School (OHFS), part of the Master of Science in Global Health Delivery program [5]. This hands-on field course provides students with practical experience that cannot be replicated in a classroom, engaging them directly with real-world scenarios to understand the complexity of health issues at the One Health interface.

One Health is an approach that is increasingly being recognized and applied worldwide. It emphasizes the interconnection between human, animal, and environmental health, fostering collaborative efforts across various disciplines to achieve optimal health outcomes [6]. This approach acknowledges that human health is intricately linked to the health of animals and our shared environment. For instance, addressing livestock-associated antimicrobial resistance, which is predicted to cause over 10 million deaths annually by 2050, requires such a comprehensive approach [7].

The current education system often fails to equip health professionals to address twenty-first century problems, highlighting a critical gap in addressing these multifaceted health issues effectively [8]. While some progress has been made in integrating global health into health professions training, gaps persist [9,10]. To realize this vision of a health professional for a new century, instructional and institutional reforms are essential, guided by transformative learning experiences. Transformative learning evolves from the acquisition of knowledge to the cultivation of leadership qualities, emphasizing shifts from rote memorization to informed decision-making, mastering core competencies, and creatively utilizing global resources [8].

The One Health Joint Plan of Action (2022–2026) is an innovative collaborative commitment developed by four key international organizations: the Food and Agriculture Organization, the United Nations Environment Programme, the World Health Organization, and the World Organisation for Animal Health [11]. The plan emphasizes this need for capacity building and educational initiatives to enhance the understanding and implementation of One Health. This approach is exemplified by UGHE's OHFS, which fosters hands-on learning and teamwork to tackle complex health issues.

## The One Health Field School

The 2-week OHFS is a transformative educational experience designed to equip students with the knowledge and skills needed to understand and act on the interconnectedness between human, animal, and environmental health. Table 1 provides an example of the OHFS schedule which takes place in varied settings such as rural communities, rice fields, national parks, health centers, and classrooms. This exposure to different environments helps students become more adaptable as they learn to navigate new and unfamiliar situations. The learning objectives of the OHFS module are designed to provide learners with a comprehensive understanding of One Health and its applications. Learners will identify the principles of One Health across various settings, including urban, rural, and park environments, and apply these principles and tools effectively. Emphasizing leadership and strategic thinking, the module helps learners define problems and set priorities for improvement. Additionally, participants will develop skills to use data for identifying, prioritizing, designing, and evaluating the impact of interventions. Effective communication is a key focus, ensuring the ability to engage with stakeholders in both lay and scientific language. The module also explores the opportunities and challenges of applying the One Health approach in real-world scenarios, encouraging innovative strategies for promoting healthy coexistence between humans and animals in sustainable local ecosystems. Lastly, learners will gain insights into evidence-based implementation, enabling them to translate research into practice effectively.

**Table 1.** Example schedule for the One Health Field School (OHFS), highlighting key activities and themes across sessions.

Day	Topic	Activities
Week 1		
Day 1	Introduction & Deployment to the Field	- Students are provided with course introduction - Students are given basic skills on using GIS methods for mapping - Students travel to the field and groups are formed
Day 2	Animal Health & Livestock Disease Surveillance	- Abattoir-related work: Visit, data collection, discussions with site-actors - Debriefing session and discussions among students and mentors - GIS course continues
Day 3	Animal Health & Veterinary Services	- National Veterinary Services (VS) is visited: Data collection from laboratory tests, discussions with actors in the various units of the VS - Debriefing session and discussions among students and mentors
Day 4	Human Health - Neglected Tropical Disease (NTDs)	- Visit Health Center treating podoconiosis: Discussions with patients and health care providers on the causes and consequences of the condition and available control measures and associated challenges. - Debriefing session and discussions among students and mentors
Day 5	Animal Health - Wildlife	- Golden Monkey tracking: Recognize the interface of wildlife, humans and livestock at the edge of the park; Discussions with the park staff, villagers about conservation and co-existence challenges. - Debriefing session and discussions among students and mentors
Day 6	Group Presentations & Departure	- Group presentations and feedback from students’ groups
Week 2		
Day 7	Travel & Introduction	- Students are provided with course introduction - Students travel to the field and group are formed
Day 8	Environmental Health	- Visit of a mining site: Appreciate mining-related environmental degradation and available minimization strategies, waste management and possible contamination, as well as occupational hazards linked to the mining activities. - Debriefing session and discussions among students and mentors
Day 9	Food Safety	- Visit to a Milk Collection Center and Processing Factory: Discussion about potential food safety relevant for milk and milk products and appreciate role of various stakeholders across the milk value chain - Debriefing session and discussions among students and mentors
Day 10	Antimicrobial Stewardship & AMR Surveillance	- Visit a referral hospital: Understand their strategy about antimicrobial stewardship and antibiotic resistance - Critically appreciate the Integrated Surveillance System implemented by Rwanda’s One Health Unit - Debriefing session and discussions among students and mentors
Day 11	Infectious Disease Management - Vector Control	- Evaluate the Community-based malaria mosquito surveillance and the field/laboratory surveillance the *Schistosoma* spp. vectors - Debriefing session and discussions among students and mentors
Day 12	Group Presentations & Departure	- Group presentations and feedback from students’ groups.

The planning and teaching strategy of the OHFS emphasizes a structured, interdisciplinary, and experiential approach to learning that integrates theoretical knowledge with practical application (see Textbox 1 for the module learning objectives). The OHFS addresses various core competencies that span (i) knowledge and understanding of current One Health complex challenges, (ii) applied knowledge and understanding of skills and techniques to develop relevant One Health interventions, (iii) taking decisions benefiting prevention and control of One Health issues even under lack of data, (iv) apply good communication using appropriate tools and techniques, and (v) appreciate the values and benefits from working as a transdisciplinary team. The competencies in the OHFS are in line with recommended competencies for One Health programming [12–14]. Teaching methods include site visits, interactive discussions, hands-on data collection, and collaborative problem-solving exercises, all aimed at encouraging critical thinking and teamwork.

For example, students visit the oldest and largest abattoir in Rwanda. Here, they observe the flow of animals and meat, identify common reasons for meat condemnation, and understand the challenges faced by abattoir workers. This visit highlights the crucial roles of veterinarians in ensuring public health by maintaining meat hygiene, authorizing meat for consumption, and collecting epidemiological data on zoonotic diseases like anthrax, salmonellosis, and brucellosis. Later in the week, students deepen their understanding by visiting the national veterinary services laboratory. They observe how abattoir sites are integrated into the surveillance system, prioritize monitored diseases, and analyze the challenges and successes in animal disease surveillance, thereby building their knowledge and resilience in tackling public health issues.

In another practical experience, students visit a mining site to document various activities, explore environmental impacts, and assess the available sanitation facilities. The environmentally aggressive mining activities, which can lead to deforestation, soil contamination, and noise pollution, present a stark learning environment. Students inquire about potential conflicts or support provided to neighboring farming communities and examine metal contamination measures. Through these experiences, they gain a comprehensive understanding of the environmental health challenges and the importance of sustainable practices in maintaining public health.

Building on Kolb's experiential learning model, the OHFS exposes students to actual field experiences and mentors them to apply their learning by designing interventions to solve identified One Health challenges [15]. Kolb's model outlines four key stages of learning: Reflective Observation, Concrete Experience, Abstract Conceptualization, and Active Experimentation. The process begins with Reflective Observation where they watch and record events and retrieve on-site retrospective data in the varied sites. This stage encourages careful analysis and awareness of the interconnected challenges in One Health. Then moving to Concrete Experience, students define One Health challenges, engage in communication and stakeholder involvement, establish drivers, and design interventions. This immersive engagement deepens their understanding of the dynamic interplay between human, animal, and environmental health. This leads to Abstract Conceptualization, where students think critically about the One Health challenges and connect the drivers. Finally, Active Experimentation involves applying their theoretical and observational insights into real-world contexts, ensuring a comprehensive understanding and effective problem-solving approach in One Health initiatives. By incorporating all stages of Kolb's learning model, the OHFS ensures that students transition from passive learners to active practitioners, equipping them with the skills to address complex One Health challenges effectively and sustainably. This integrated approach underscores the importance of linking theory with practice to foster innovative and actionable solutions.

An example of Kolb's experiential learning model in practice can be seen in the students' visit to a Milk Collection Center (MCC). In countries like Rwanda, farmers typically deliver their cows' milk to MCCs, which aggregate milk from various sources before selling it to processors and consumers. During the OHFS, students visited a rural MCC to explore the possibility of the MCC delivering poor quality and unsafe milk to users downstream of the milk value chain. Using Kolb's experiential learning model, the students witnessed how milk is delivered and stored at MCCs, fostering an understanding of milk production practices and associated challenges. Specifically, the students engaged with farmers, MCC staff, and local leaders to understand the root causes of these practices and their potential impact on human health (e.g. foodborne illnesses), animal health (e.g. mastitis in cows), and environmental health (e.g. waste management issues). This experience supports their knowledge of the hygiene around the milk supply chain and quality tests conducted at MCC to ensure safety and consumer protection. Finally, the assigned group designed interventions that included incentivizing milk producers to deliver milk under cold conditions, regular testing of antibiotics, which is not routinely done, and consumer awareness about the role of an MCC, which is not very well understood (i.e. some consumers believe milk from MCC's tank is safe for immediate consumption without pasteurization). These discussions provide a practical case study for the in-class Food Security module, enabling students to apply their knowledge to suggest interventions to improve milk quality and safety and minimize the farmers' losses due to milk rejections.

## Bridging theory to practice

The experiences gained during the OHFS are crucial for bridging the gap between classroom theory and real-world practice. Field courses challenge students to apply their classroom knowledge to solve practical problems in real-time, developing critical thinking and problem-solving skills. Students learn to navigate the complexities and uncertainties of real-world health issues, which is essential for their future roles.

Continuing their hands-on learning journey, students visit a health center in Rwanda's Northern Province that treats podoconiosis, a non-filarial elephantiasis caused by volcanic soil. They observe patient care, recruitment processes, and mental health support and educational interventions. This practical session allows students to apply classroom knowledge from the NTD module, understanding podoconiosis management and discussing its mental and socioeconomic impacts with patients.

During the OHFS, students are assigned to specific sites where they work as interdisciplinary teams to identify and analyze One Health problems. They assess potential drivers and propose interventions that benefit the health of humans, animals, and the environment. This collaborative effort enhances their communication and teamwork skills as they share responsibilities and adapt to various roles. Real-world projects often involve complexities and uncertainties, and navigating these challenges helps students build resilience and adaptability. The OHFS experience enables students to think critically, adapt to changing circumstances, and develop innovative solutions. By addressing real-world problems, students enhance their problem-solving skills and are better equipped to handle the unpredictability of practical situations, ultimately fostering their ability to lead and collaborate effectively.

## Shaping future leaders

The OHFS at UGHE is not just about imparting knowledge; it is about shaping the next generation of leaders. By fostering a holistic mindset and providing practical tools, the module prepares students to respond to health threats effectively. The OHFS stands as a model for how interdisciplinary education can lead to innovative solutions and better health outcomes.

One Health is inherently interdisciplinary, encompassing fields such as human and veterinary medicine, environmental, and public health. Field courses enable students to witness firsthand how these disciplines converge in real settings, fostering a more integrated and holistic understanding of health. This interdisciplinary exposure is crucial for preparing students to tackle multifaceted health challenges. Their teamwork strengthens communication and collaboration across various disciplines and cultural backgrounds.

The OHFS helps students learn relevant soft skills, including communication, transdisciplinary collaboration, and leadership. By working in teams, students enhance their ability to communicate effectively and collaborate with peers from diverse backgrounds and disciplines. The interactions with rural farmers, health professionals, government officials, and other stakeholders during field visits help students design and implement interventions, thereby developing essential leadership skills and ensuring a comprehensive approach to health issues by incorporating multiple perspectives. Additionally, students gain an appreciation for the value of engaging community members in the analysis and response to One Health challenges, fostering a sense of responsibility and resilience as they witness the direct impact of their work on the community [16].

Hands-on experiences in the field can inspire students to pursue careers in One Health and related fields. Seeing the tangible impact of their work on communities and environments motivates students to become leaders and changemakers in global health. This inspiration is essential for cultivating the next generation of health professionals committed to making a difference. By bridging theory and practice, the OHFS equips students with the skills needed to navigate the complexities of real-world health issues and emerge as effective leaders in the global health landscape.

## Conclusion

As we face the growing impacts of environmental degradation and other global health threats, the need for leaders trained in the One Health approach is more critical than ever. By embracing this interdisciplinary approach, UGHE is leading the way in training future leaders who can navigate the complexities of modern health challenges and drive positive change in their communities and beyond.

Field-based education teaches students to adapt to changing circumstances and work in diverse settings. These experiences build resilience and adaptability, which are important traits for professionals working in dynamic and challenging environments. Adaptability and resilience are crucial for responding effectively to evolving health threats. Students learn to seek out new experiences, embrace challenges, and continually adapt to new information and environments.

The OHFS at UGHE has seen success, with alumni actively contributing to their organizations and leading impactful projects. For instance, one alumnus heads the One Health Unit in the Government of Rwanda, while another is conducting a research project on cysticercosis, an infection caused by the larval cysts of the pork tapeworm, which affects both humans and animals and can lead to serious health issues. These examples demonstrate the real-world impact of their training. However, further comprehensive assessment should be carried out to determine if and how the alumni are using the acquired skills in their current employment.

In conclusion, a hands-on, field-based course in One Health is essential for providing comprehensive education that equips students with the knowledge, skills, and experiences needed to address the complex and interconnected health challenges of our time. By bridging the gap between theory and practice, fostering interdisciplinary learning, and promoting community engagement, these courses prepare students to become effective leaders and changemakers in global health.

## References

[1] Jones KE, Patel NG, Levy MA, et al. Global trends in emerging infectious diseases. Nature. 2008;451:990–6. doi: 10.1038/nature06536 18288193 PMC5960580

[2] International Livestock Research Institute. Preventing and controlling human diseases transmitted by animals saves millions of lives and livelihoods. Livestock pathways to 2030: one health brief 2. International Livestock Research Institute; 2021 [cited 2024 Dec 3]. Available from: https://cgspace.cgiar.org/server/api/core/bitstreams/58676769-0e31-43e3-9045-e70bfb65bf77/content

[3] World Health Organization. Estimates of the global burden of foodborne diseases: foodborne disease burden epidemiology reference group 2007–2015. Geneva: World Health Organization; 2015 [cited 2024 Dec 3]. Available from: https://iris.who.int/bitstream/handle/10665/199350/9789241565165_eng.pdf?sequence=1

[4] Ministry of Health | Rwanda. Amakuru mashya kuri Virusi ya Marburg. Update on Marburg Virus Disease 31.10.2024 [Tweet]. 2024 Oct 31 [cited 2024 Dec 3]. Available from: https://x.com/RwandaHealth

[5] University of Global Health Equity. One health field school 2022 highlights [Internet]. YouTube. 2022 May 22 [cited 2024 Dec 3]. Available from: https://www.youtube.com/watch?v=wAgOKf8Rwic

[6] Mettenleiter TC, Markotter W, Charron DF, et al. The one health high-level expert panel (OHHLEP). One Health Outlook. 2023;5. doi: 10.1186/s42522-023-00085-2 PMC10704771 38062534

[7] O’Neill J. Review on antimicrobial resistance. Tackling drug-resistant infections globally: final report and recommendations. Review on antimicrobial resistance. 2016.

[8] Frenk J, Chen L, Bhutta ZA, et al. Health professionals for a new century: transforming education to strengthen health systems in an interdependent world. Lancet. 2010;376:1923–1958. doi: 10.1016/S0140-6736(10)61854-5 21112623

[9] Carrasco H, Fuentes P, Eguiluz I, et al. Evaluation of a multidisciplinary global health online course in Mexico. Glob Health Res Policy. 2020;5. doi: 10.1186/s41256-020-00179-8 PMC7670103 33292748

[10] Martin MH, Rose ES, Jahangir E, Heimburger DC. Ten-year evaluation of an immersive global health medical school course using a four-principle equity framework. Front Educ. 2023;8. doi: 10.3389/feduc.2023.1200389

[11] Food and Agriculture Organization of the United Nations, United Nations Environment Programme, World Health Organization, World Organisation for Animal Health. One health joint plan of action (2022–2026): working together for the health of humans, animals, plants and the environment. Geneva: World Health Organization; 2022 [cited 2024 Nov 8]. Available from: https://iris.who.int/bitstream/handle/10665/363518/9789240059139-eng.pdf?sequence=1

[12] Laing G, Duffy E, Anderson N, et al. Advancing one health: updated core competencies. CABI One Health. 2023. doi: 10.1079/cabionehealth.2023.0002

[13] Togami E, Gardy JL, Hansen GR, et al. Core competencies in one health education: what are we missing? NAM Perspect. 2018;8. doi: 10.31478/201806a

[14] The Inter-University Council for East Africa and International Livestock Research Institute. Benchmarks for master in one Heath. 2024 [cited 2024 Feb 4]. Available from: https://onehealthobservatory.org/resources/benchmarks-master-one-health

[15] Kolb DA. Learning styles and disciplinary differences. In: Chickering A, editor. The modern American college. San Francisco: Jossey-Bass; 1981. p. 232–255.

[16] Henley P, Igihozo G, Wotton L. One health approaches require community engagement, education, and international collaborations—a lesson from Rwanda. Nat Med. 2021;27:947–948. doi: 10.1038/s41591-021-01350-5 34017132

